# Metabolic Response of *Candida albicans* to Phenylethyl Alcohol under Hyphae-Inducing Conditions

**DOI:** 10.1371/journal.pone.0071364

**Published:** 2013-08-12

**Authors:** Ting-Li Han, Sergey Tumanov, Richard D. Cannon, Silas G. Villas-Boas

**Affiliations:** 1 Centre for Microbial Innovation, School of Biological Sciences, The University of Auckland, Auckland, New Zealand; 2 Department of Oral Sciences, University of Otago, Dunedin, New Zealand; Imperial College London, United Kingdom

## Abstract

Phenylethyl alcohol was one of the first quorum sensing molecules (QSMs) identified in *C. albicans*. This extracellular signalling molecule inhibits the hyphal formation of *C. albicans* at high cell density. Little is known, however, about the underlying mechanisms by which this QSM regulates the morphological switches of *C. albicans*. Therefore, we have applied metabolomics and isotope labelling experiments to investigate the metabolic changes that occur in *C. albicans* in response to phenylethyl alcohol under defined hyphae-inducing conditions. Our results showed a global upregulation of central carbon metabolism when hyphal development was suppressed by phenylethyl alcohol. By comparing the metabolic changes in response to phenylethyl alcohol to our previous metabolomic studies, we were able to short-list 7 metabolic pathways from central carbon metabolism that appear to be associated with *C. albicans* morphogenesis. Furthermore, isotope-labelling data showed that phenylethyl alcohol is indeed taken up and catabolised by yeast cells. Isotope-labelled carbon atoms were found in the majority of amino acids as well as in lactate and glyoxylate. However, isotope-labelled carbon atoms from phenylethyl alcohol accumulated mainly in the pyridine ring of NAD^+^/NADH and NADP^−/^NADPH molecules, showing that these nucleotides were the main products of phenylethyl alcohol catabolism. Interestingly, two metabolic pathways where these nucleotides play an important role, nitrogen metabolism and nicotinate/nicotinamide metabolism, were also short-listed through our previous metabolomics works as metabolic pathways likely to be closely associated with *C. albicans* morphogenesis.

## Introduction

In *C. albicans*, the switch from yeast to hyphal growth or *vice versa* is determined by environmental signals that trigger signal transduction pathways and change gene expression. The environmental perturbations can arise from modifications in the level of nutrients, or from molecules that are secreted by *C. albicans* in a cell density-dependent fashion known as quorum sensing molecules (QSMs) [1]. There are a number of QSMs which have been identified in *C. albicans*, including farnesol, farnesoic acid, tyrosol, tryptophol, and phenylethyl alcohol [2]–[5].

Phenylethyl alcohol was one of the first QSMs to be identified in *C. albicans* [4]. This molecule inhibits both filamentous growth and germ tube formation of *C. albicans* once its extracellular concentration reaches a certain threshold value. On the other hand, Chen & Fink [6] demonstrated that phenylethyl alcohol has an opposite effect in *S. cerevisiae,* where it stimulates filamentous growth in response to ammonia starvation and high cell density. These authors proposed that phenylethyl alcohol is involved in nitrogen- and signalling-mediated morphogenesis in *S. cerevisiae*. They suggested that ammonia starvation triggers morphogenesis by regulating the production of phenylethyl alcohol which increases in concentration because the reduced amount of ammonium ion alleviates repression of *ARO9*, *ARO10*, and other genes required for aromatic alcohol production. Phenylethyl alcohol is also thought to promote *S. cerevisiae* morphogenesis by upregulating an essential filamentous gene, *FLO11,* via a PKA pathway-dependent mechanism [6]. In comparison, little is known about the molecular mechanisms by which phenylethyl alcohol regulates the morphological switch of *C. albicans*. What is known is that the extracellular concentration of phenylethyl alcohol increases in the presence of phenylalanine supplementation in the growth medium, at low ammonia concentration, under alkaline pH and under anaerobic conditions [7]. In addition, phenylethyl alcohol production is not affected by alkaline pH when the transcriptional regulators, *ARO80* and *RIM101* genes, are disrupted [7].

Therefore, to further investigate the metabolic response of *C. albicans* to phenylethyl alcohol, we have applied a gas chromatography-mass spectrometry (GC-MS) metabolomics approach, and isotope labelling experiments, under hyphae-inducing conditions. *C. albicans* is one of the most prevalent pathogenic yeast species in humans that can cause candidiasis at multiple sites, from mucosae to internal organs. Therefore, understanding the metabolic mechanisms behind one of its key virulence traits, morphogenesis, may provide insights for novel therapeutic interventions to prevent *C. albicans* infections.

## Materials and Methods

### Chemicals

Methanol, chloroform, sodium bicarbonate, and sodium hydroxide were obtained from MERCK (Darmstadt, Germany). The internal standard 2,3,3,3-d_4_-alanine, the derivatization reagent methyl chloroformate (MCF), pyridine, and *D*-glucose-^13^C_6_ were purchased from Sigma-Aldrich (St. Louis, USA). Anhydrous sodium sulphate and pheneylethyl alcohol were obtained from Fluka (Steinheim, Germany). All chemicals were of analytical grade.

### Fungal Strain and Culture Media

*C. albicans* strain SC5314 [8] was maintained on YPD agar medium containing yeast extract (6 g/L), peptone (3 g/L), glucose (10 g/L), and agar (15 g/L); at 30°C. Pre-inocula were prepared in minimum mineral medium (MM medium) at pH 5.5 containing D-glucose (10 g/L), (NH_4_)_2_SO_4_ (5 g/L), MgSO_4_⋅7H_2_O (0.5 g/L), KH_2_PO_4_ (3 g/L), vitamins and trace metals as previously described [9]. Three different culture media were used for the metabolomic and isotope labelling experiments. (1) Minimum mineral medium (MM medium) at pH 7.5; (2) phenylethyl alcohol medium (PA medium), which consisted of MM medium supplemented with phenylethyl alcohol (1.5 mM); and (3) phenylethyl alcohol medium with ^13^C-labelled glucose (PA ^13^C-glucose medium), which consisted of MM medium supplemented with phenylethyl alcohol (1.5 mM) and with glucose (10 g/L) uniformly labelled with ^13^C instead of ^12^C-glucose.

### Culture Conditions

*C. albicans* was cultured in 250 mL MM medium (pH 5.5) at 30°C using shake flasks in a rotary shaker overnight. The cells were collected by centrifugation at 2000 *g* (4°C) for 5 min and washed in phosphate buffered saline (8 g/L NaCl, 0.2 g/L KCl, 1.44 g/L Na_2_PO_4_, 0.24 g/L KH_2_PO_4_, at pH 7.5). The cells were resuspended in the three different growth media described above, at an initial OD_600_ of 0.2. The cells were incubated in a rotary shaker-incubator at 37°C for 12 hr. The morphology of *C. albicans* cells in each growth medium was monitored using a phase contrast microscope (DMR, Lecia).

### Sampling and Quenching of Cell Metabolism

Five replicate shake-flask cultures (30 mL) for each growth medium were harvested at middle to late exponential growth phase (12 hours). Samples (2 mL) of the microbial cultures were membrane-filtered (0.2 µm) to remove *C. albicans* cells, and the filtrate was used for the analysis of extracellular metabolites. The remaining 28 mL of culture were rapidly filtered under vacuum (Air Cadet vacuum/pressure station, Thermo), quickly washed with cold phosphate buffered saline solution (1–2°C) and quenched in cold methanol water (1∶1_v/v_) at −30°C as described by Smart *et al*. [10]. The whole sampling procedure took less than 30 s per sample.

### Sample Preparation for Metabolite Analysis

The intracellular metabolites were extracted from the quenched cell pellets using cold methanol water and freeze-thaw cycles following the protocol described by Smart *et al*. [10]. The Internal standard 2,3,3,3-d_4_-alanine (0.3 µmol/sample) was added to each sample before extraction. The intracellular metabolite extracts and 1 mL of spent culture medium containing extracellular metabolites were freeze-dried (BenchTop K manifold freeze dryer, VirTis) before chemical derivatization.

### Chemical Derivatization of Metabolites

The freeze-dried samples were derivatized using the methyl chloroformate (MCF) protocol developed in-house and described in Smart *et al*. [10].

### Gas Chromatography-Mass Spectrometry (GC-MS) Analysis

The MCF derivatives were analysed in an Agilent GC7890 system coupled to a MSD5975 mass selective detector (EI) operating at 70 eV. The column used for all analyses was a ZB-1701 GC capillary column (30 m × 250 µm id × 0.15 µm with 5 m guard column, Phenomenex). The GC-MS parameters were set according to Smart *et al.* [10]. Samples were injected under pulsed splitless mode with the injector temperature at 290°C. The helium gas flow through the GC-column was set at 1.0 mL/min. The interface temperature was set to 250°C and the quadrupole temperature was 200°C.

### Biomass Quantification

The cell debris collected after intracellular metabolite extraction was dried using a domestic microwave (250 W for 20 min) and weighed in order to measure the total biomass content (dry weight) of each sample.

### Data Mining, Data Normalization, and Data Analysis

AMDIS software (NIST, Boulder, CO, USA) was used for deconvoluting GC-MS chromatograms and identifying metabolites using our in-house MCF MS library. The identifications were based on both the MS spectrum of the derivatized metabolite and its respective chromatographic retention time. The relative abundance of identified metabolites was determined by ChemStation (Agilent) using the GC base-peak value of a selected reference ion. These values were normalized by the biomass content in each sample as well as by the abundance of internal standard (2,3,3,3-d_4_-alanine). A univariate analysis of variance (ANOVA) was applied to determine whether the relative abundance of each identified metabolite was significantly different between growth conditions. Our Pathway Activity Profiling (PAPi) algorithm [11] was used to predict and compare the relative activity of different metabolic pathways in *C. albicans* during the growth conditions tested based on metabolite profiling results. This programme connects to the KEGG online database (http://www.kegg.com) and uses the number of metabolites identified from each pathway and their relative abundances to predict which metabolic pathway is likely to be active in the cell. The entire data mining, data normalization and pathway activity predictions were automated in R software as described in Smart *et al*. [10] and Aggio *et al*. [11]. Graphical representations of the results were generated by gplots and ggplot2 R packages [12], [13].

### Analysis of Isotope Labelling Distribution in the Detected Metabolites

Due to the commercial unavailability of isotopically labelled phenylethyl alcohol, we decided to apply an inverse isotope labelling approach whereby all *C. albicans* metabolites were fully labelled with ^13^C by culturing them in MM medium with ^13^C-U-labelled glucose as the sole carbon source. This way, we searched for ^12^C-enrichment in the metabolite profile originating from the metabolism of ^12^C-phenylethyl alcohol. First, we identified the ^13^C-labelled metabolites based on their chromatographic retention times obtained by GC-MS analysis of metabolite extracts of cells grown under the same conditions but with ^12^C-glucose as sole carbon source. The electron-impact fragmentation pattern of each identified MCF derivatized ^13^C-labelled metabolite was determined based on its corresponding ^12^C spectrum from our MS library. The degree of labelling was estimated based on the variation observed in the mass spectrum fragmentation pattern of fully ^13^C-labelled metabolites in comparison to their counterpart ^12^C mass spectrum.

## Results

### Suppression of Hyphal Formation by Phenylethyl Alcohol

**Figure 1:**
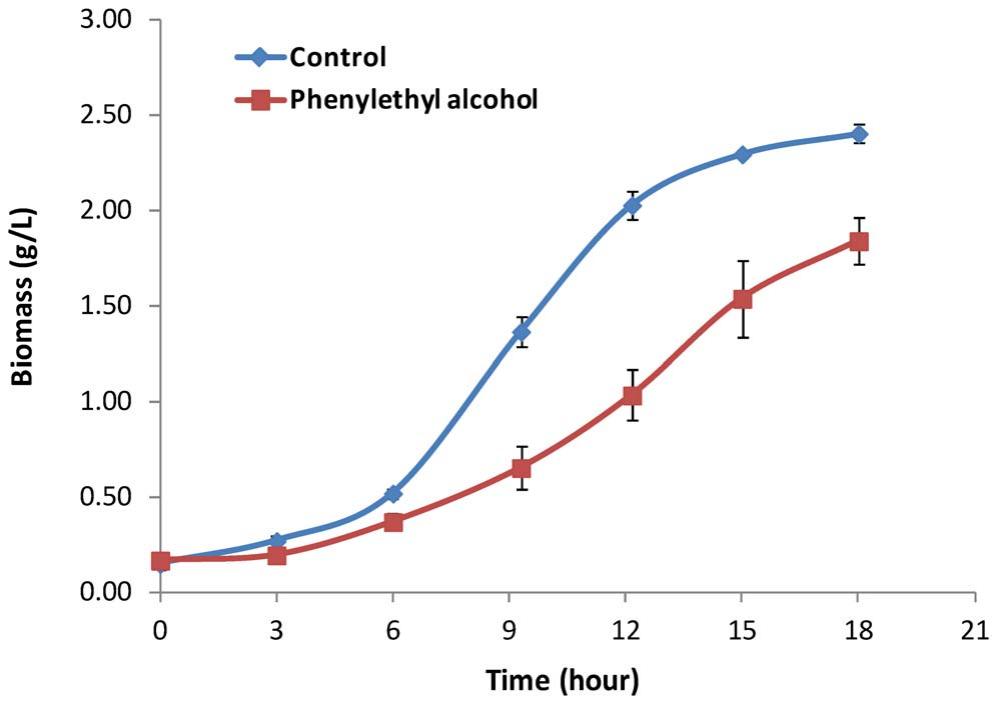
The morphology of *C. albicans* cells incubated in the presence of different concentrations of phenylethyl alcohol in minimal mineral medium (MM) at 37°C for 12 h. The Images were taken by Nomarksi contrast microscopy with 800× magnification.

**Figure 2:**
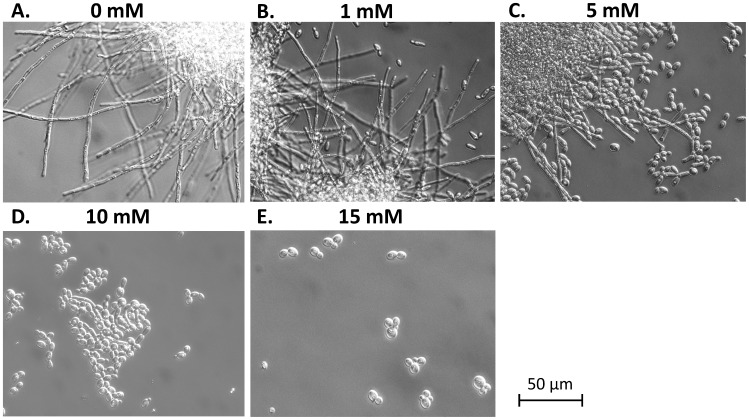
Growth curves of *C. albicans* cells grown in the presence (red) or absence (blue) of phenylethyl alcohol (15 mM).

*C. albicans* morphogenesis was completely inhibited by 15 mM pheneylethyl alcohol (Figure 1). Phenylethyl alcohol (15 mM) reduced both the growth rate and biomass yield of *C. albicans* (Figure 2, Table 1) but did not kill the cells. At the sampling point (t = 12 h), microscopic examination showed that phenylethyl alcohol (15 mM) completely inhibited hyphal formation under the growth condition tested, whilst at least 95% of cells grew as hyphae when phenylethyl alcohol was not present (Figure 1).

**Table 1:** Biomass and morphology of *C. albicans* cells cultured in different growth media for 12 h.

Media (pH 7.4, 37°C)	Main carbonsource	Biomass(mg/mL)	F (%)
MM	^12^C-glucose	2.03	>95
MM+Phenylethyl alcohol	^12^C-glucose	0.82	0
MM+Phenylethyl alcohol	^13^C-glucose	0.72	0

MM: minimum mineral medium; F: percentage of filamentous growth determined by counting the number of yeast cells and filaments in 1 mm^3^ volume.

### The Extracellular Metabolite Profile of *C. albicans* Under Suppression of Hyphae Formation by Phenylethyl Alcohol

**Figure 3:**
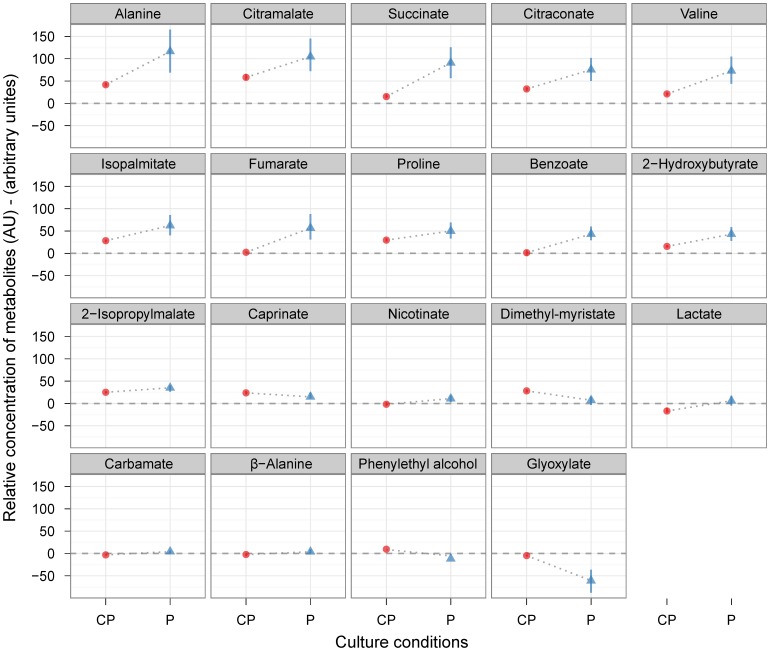
Relative concentrations of extracellular metabolites after 12 hours of incubation in the presence or absence of phenylethyl alcohol. Phenylethyl alcohol treatment (P) is represented by blue triangles and control (C) hyphae-inducing conditions (MM) are represented by red circles. The concentrations of identified metabolites have been normalised by internal standard (d_4_-alanine) and biomass before the relative concentrations of the corresponding metabolites found in un-inoculated culture medium were subtracted. Standard deviations are indicated by the vertical line-range. The difference between secretion and consumption of extracellular metabolites is distinguished by the dashed lines (y = 0). Secretion of metabolites is indicated by positive values. Consumption of metabolite is indicated by negative values. Only the metabolites generating statistically significant ANOVA scores (p-value<0.05) are shown.

We detected over 50 metabolites in the spent culture samples of *C. albicans* and we were able to accurately identify 26 of them using our in-house MS library, including phenylethyl alcohol (Table 2). Nineteen metabolites were detected at significantly different levels when comparing MM and phenylethyl alcohol-supplemented MM cultures (Figure 3). Of these extracellular metabolites 16 appear to have been secreted by *C. albicans* cells in phenylethyl alcohol supplemented cultures. These metabolites included alanine, *β*-alanine, benzoate, carbamate, citraconate, citramalate, fumarate, isopalmitate, lactate, nicotinate, proline, succinate, valine, 2-hydroxybutyrate, and 2-isopropylmalate. Other compounds such as glyoxylate seem to have been taken up more extensively by *C. albicans* cells growing in the presence of phenylethyl alcohol, because they were detected at significant lower concentration compared to the MM medium without phenylethyl alcohol. Interestingly, control cultures without phenylethyl alcohol supplementation secreted small amounts of phenylethyl alcohol to the extracellular medium and cultures with phenylethyl alcohol supplementation showed 16% reduction in the extracellular phenylethyl alcohol levels when compared to non-inoculated medium, therefore confirming that *C. albicans* cells took up phenylethyl alcohol during growth.

**Table 2:** Intracellular and extracellular metabolites associated with *C. albicans* growth in different culture media.

Classification of metabolites	Intra	Extra	Metabolites
Amino acids	19	5	Alanine*, asparagine, aspartate, cysteine, glutamine, glutamate, glycine*, histidine, isoleucine, leucine, lysine, phenylalanine, proline*, serine, threonine, tryptophan, tyrosine, valine*, β-alanine*
Amino acid derivatives	9	0	Creatinine, cystathionine, D-2-aminoadipiate, *N*-acetylglutamate, norvaline, ornithine, S-adenosyl-L-homocysteine, 2-aminobutyrate, and pyroglutamate
TCA cycle intermediates	7	3	Fumarate*, citrate*, succinate*, *cis*-aconitate, isocitrate, malate, 2-oxoglutarate*
Fatty acids	12	4	Adipate, Caprinate*, caprylate, D-2-aminoadipate, lactate*, malonate, myristate, oleate, pentadecanoate, isopalmitate*, stearate*, and 3-hydroxyoctanoate
Glycolytic intermediates	2	0	Pyruvate and phosphoenolpyruvate.
Cofactors and vitamins	3	1	NADP/NADPH, nicotinate*, and 4-amino-*n*-butyrate
Others	15	10	Benzoate*, cabamate*, citraconate*, citramalate*, eichosanoate, glutarate*, itaconate, lactate, malonate, 2-isopropylmalate*, 2-hydroxybutyrate*, dimethyl- myristate*, 3,5-bis(1,1-dimethylethyl)-4-hydroxy-benzenepropanoic acid*, 4- aminobenzoate 10,12-octadecadienoate, and phenylethyl alcohol*
Metabolites only found in extracellular media	0	3	Glyoxylate/glyoxalate*, 12-oxoglutarate*, 2,4-bis(1,1-dimethylethyl)-phenol*
**Total number of identified** **metabolites**	67	26	

Intra: Number of intracellular metabolites identified in any samples; Extra: Number of extracellular metabolites identified in any samples;

* Metabolites found in all extracellular media.

### The Intracellular Metabolite Profiles of *C. albicans* Under Suppression of Hyphae Formation by Phenylethyl Alcohol

**Figure 4:**
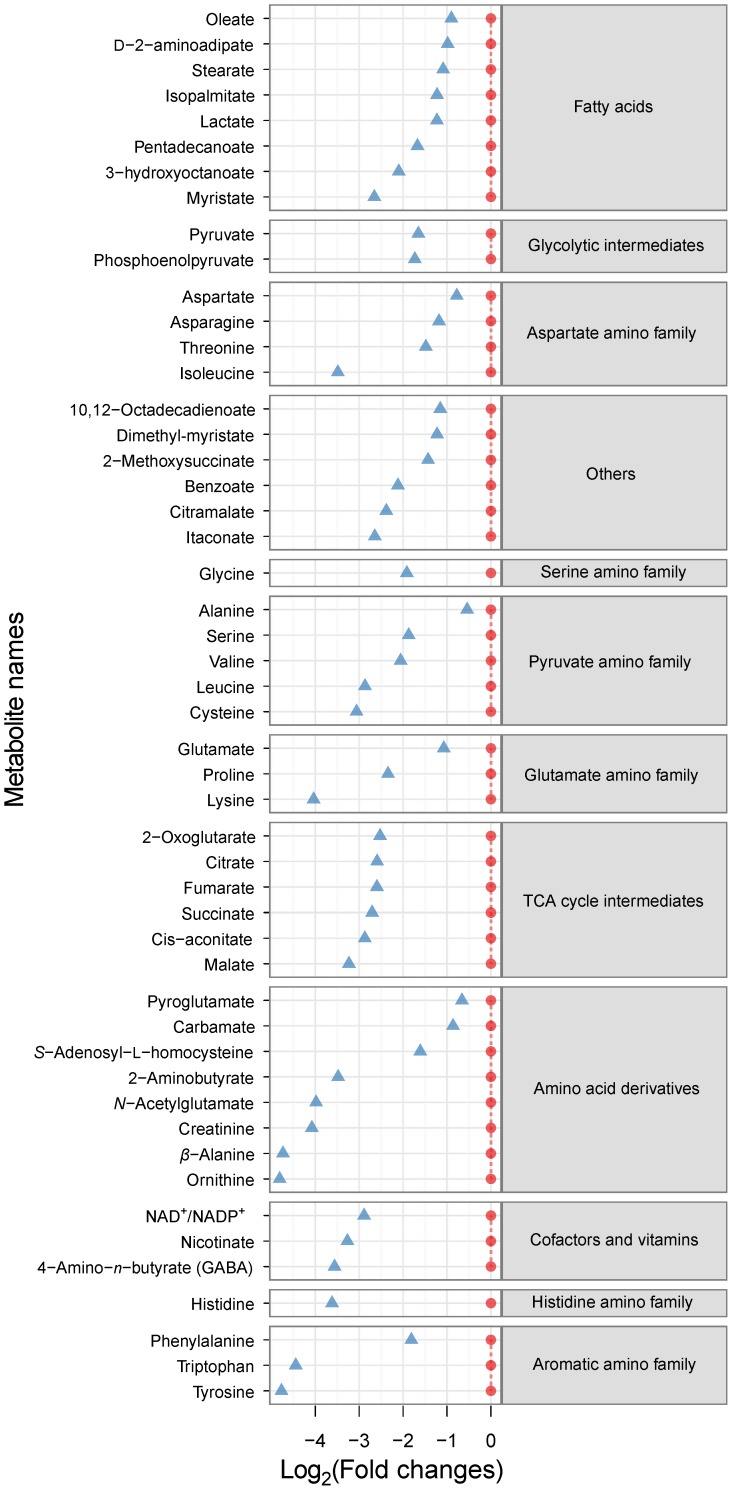
The ratio of Intracellular metabolite concentrations for cells grown in the presence of phenylethyl alcohol relative to those in cells grown in the absence of phenylethyl alcohol after 12 h incubation. Red circles represent concentrations for samples from cells incubated in the absence of phenylethyl alcohol (hyphae-inducing conditions) - that were set to 0. Blue triangles indicate metabolite concentrations in cells exposed to phenylethyl alcohol relative to those in cells grown without phenylethyl alcohol. The metabolite levels relative to the hyphal samples have been plotted using a log_2_ scale. Negative values indicate that the metabolite concentrations were reduced in response to phenylethyl alcohol. Only the metabolites generating statistically significant ANOVA scores (p-value<0.05) are shown.

In order to investigate further how *C. albicans* cells respond to phenylethyl alcohol, we compared the profiles of intracellular metabolites from cells grown in MM medium with and without phenylethyl alcohol. We detected over 100 metabolites in the intracellular metabolite extracts and 67 of them were accurately identified across samples. Of these, the concentrations of 51 metabolites were significantly different in cells grown in the phenylethyl alcohol-containing medium (Table 2, Figure 4). Interestingly, all intracellular metabolites were detected at lower concentrations in samples from cultures supplemented with phenylethyl alcohol. These intracellular metabolites include a range of intermediates from the central carbon metabolism such as amino acids, organic acids, fatty acids, and nucleotides. In particular, the concentrations of aromatic amino acids, tyrosine and tryptophan, ornithine, *β*−alanine, and lysine were reduced up to 16-fold in cells growing in phenylethyl alcohol medium. Furthermore, metabolites such as alanine, *β*-alanine, benzoate, carbamate, citramalate, lactate, nicotinate, proline, succinate, and valine were found at reduced concentrations intracellularly, but increased concentrations extracellularly in cells exposed to phenylethyl alcohol, suggesting that the cells actively secreted those metabolites when growing in culture media supplemented with phenylethyl alcohol.

### Prediction of the Metabolic State of *C. albicans* Under Suppression of Hyphal Formation by Phenylethyl Alcohol

**Figure 5:**
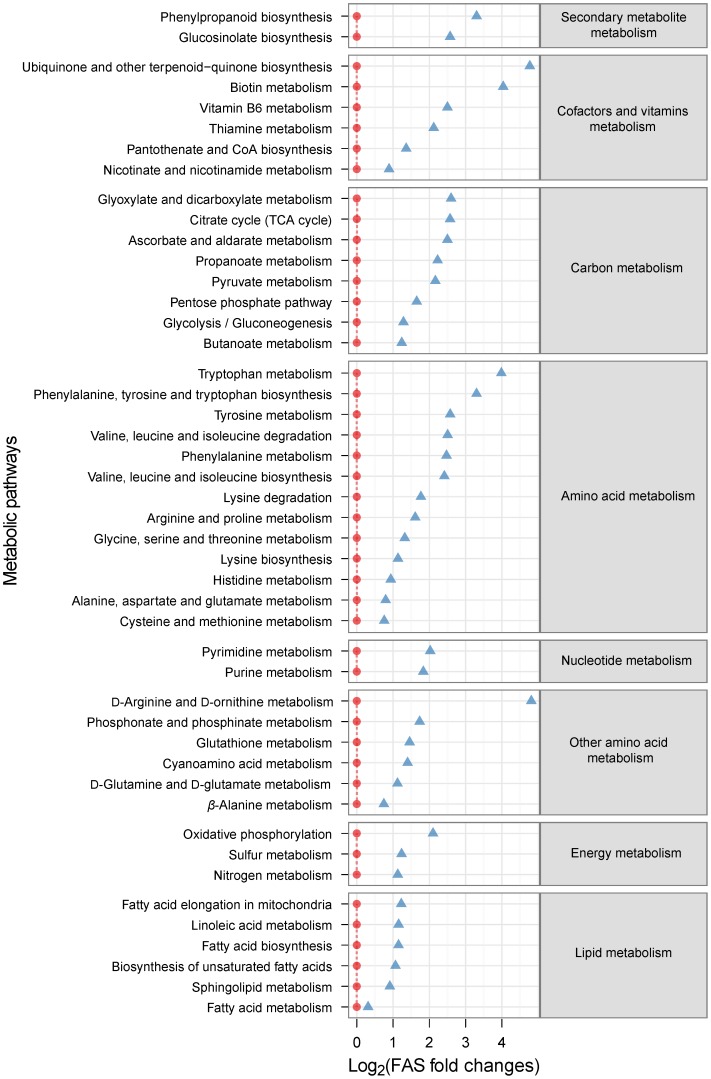
Activities of *C. albicans* metabolic pathways based on intracellular metabolomic data when cultivating *C. albicans* for 12 h in the presence or absence of phenylethyl alcohol. Red circles represent metabolic activities under hyphae-inducing conditions that were set to 0. Blue triangles indicate metabolic activities in cells treated with phenylethyl alcohol. The metabolic activities relative to the hyphae-inducing samples have been plotted using a log_2_ scale. Positive values indicate the metabolic pathways had their activity up-regulated in response to phenylethyl alcohol. Only the pathways generating statistically significant ANOVA scores (p-value<0.05) are shown.

Using the profile of intracellular metabolites identified in the samples of *C. albicans* cells growing in the presence or absence of phenylethyl alcohol, we created a comparative metabolic activity profile using PAPi software [11]. The metabolic activities of *C. albicans* cells under conditions suppressing hyphal formation by phenylethyl alcohol were compared with those in cells cultured in the absence of phenylethyl alcohol (Figure 5). All of the 48 metabolic pathways that showed significant changes in metabolic activity in the cells growing in phenylethyl alcohol-supplemented medium were up-regulated. These include a range of metabolic pathways from the metabolism of amino acids, carbon, cofactors, energy, lipids, nucleotides, and secondary metabolites. In particular, ubiquinone biosynthesis, tryptophan metabolism, and D-arginine/D-ornithine metabolism exhibited a marked upregulation probably in response to phenylethyl alcohol. This is in agreement with our metabolite profile results because two of the main assumptions of the PAPi software, which is a hypothesis generating tool, is that the higher the flux through a given metabolic pathway the larger the number of detected intermediates from that pathway will be and, most importantly, the lower will be the concentration of those compounds inside the cell, because we believe that if a pathway is operating at a high flux there will be a high turn-over rate between its intermediates and a higher incorporation of those intermediates and end products into the biomass, reducing their intracellular concentration.

### ^12^C-label Distribution Through the Metabolite Profile of *C. albicans* Grown in ^13^C-U-glucose Medium Supplemented with ^12^C-phenylethyl Alcohol

**Figure 6:**
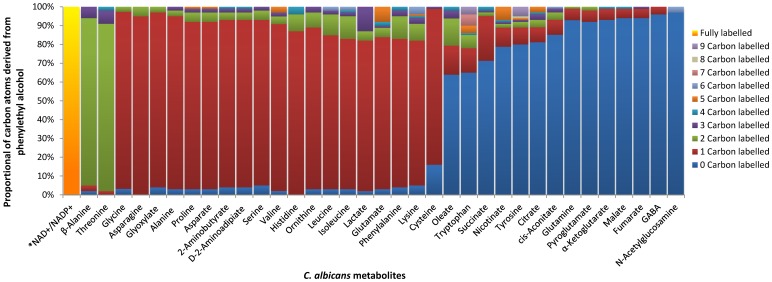
The incorporation of carbon atoms from phenylethyl alcohol into *C. albicans* metabolites under hyphae-inducing conditions. Stacked column plot indicates the percentage that the number of carbons derived from phenylethyl alcohol contributes to the total ion mass in each identified metabolite (see Table 3). Only the pyridine ring of NADPH and NADH are considered for those molecules.

When cells grown in ^13^C-glucose were exposed to ^12^C-phenylethyl alcohol, a number of metabolites contained at least one carbon from phenylethyl alcohol (Table 3). These metabolites included a number of amino acids (alanine, asparagine, asparate, cysteine, glutamate, histidine, isoleucine, leucine, lysine, ornithine, phenylalanine, proline, serine, valine, 2-aminobutyrate, D-2-aminoadipiate), glyoxylate, and lactate. The majority of threonine and β-alanine molecules contained at least two ^12^C-atoms. However, we found that the pyridine ring from the NADP^+^/NADPH and NAD^+^/NAD molecules were almost fully labelled with ^12^C in ^13^C-cultures supplemented with ^12^C-phenylethyl alcohol (Figure 6). In contrast, intermediates from the TCA cycle (citrate, cis-aconitate, α-ketoglutarate, fumarate, malate, and succinate), two aromatic amino acids (tryptophan, tyrosine), GABA, N-acetylglucosamine, nicotinate, oleate, and pyroglutamate showed no significant ^12^C-enrichment.

**Table 3:** List of ion clusters used to determine the pattern of isotope labelling in the identified metabolites.

Metabolites	Ion cluster	Carbon fragment	Mass isotopomer % abundance									
			M	M+1	M+2	M+3	M+4	M+5	M+6	M+7	M+8	M+9
2-aminobutyrate	116	C1–C4	1%	2%	4%	89%	4%					
Alanine	103	C1–C3	1%	2%	92%	4%						
Asparagine	127	C1–C4	0%	0%	5%	94%	0%					
Asparate	160	C1–C4	2%	1%	8%	86%	4%					
*Cis*-Aconitate	153	C1–C6	1%	0%	0%	2%	4%	8%	86%			
Citrate	143	C1–C5	3%	1%	3%	4%	8%	82%				
Cysteine	192	C1–C3	1%	0%	83%	16%						
D-2-aminoadipiate	188	C1–C6	1%	4%	3%	2%	7%	81%	4%			
Fumarate	113	C1–C4	0%	1%	0%	5%	94%					
GABA	144	C1–C4	0%	0%	0%	4%	96%					
Glutamate	174	C1–C5	8%	2%	1%	5%	80%	3%				
Glutamine	141	C1–C5	0%	0%	0%	1%	6%	92%				
Glycine	88	C1–C2	3%	94%	3%							
Glyoxylate	103	C1–C2	3%	93%	4%							
Histidine	226	C1–C6	0%	0%	4%	0%	9%	87%	0%			
Isoleucine	144	C1–C6	2%	0%	1%	2%	12%	80%	3%			
Lactate	103	C1–C3	13%	5%	80%	2%						
Leucine	144	C1–C6	2%	0%	0%	2%	11%	81%	3%			
Lysine	142	C1–C6	4%	1%	1%	3%	9%	77%	5%			
Malate	113	C1–C4	1%	0%	0%	5%	94%					
*N*-acetylglucosamine	158	C1–C6	3%	0%	0%	0%	0%	0%	97%			
NADP^+^/NADPH	138, 94,80	Pyridine ring of nicotinamide >95%										
NAD^+^/NADH	171,140,124	Pyridine ring of nicotinamide >95%										
Nicotinate	106	C1–C6	7%	1%	1%	2%	10%	78%				
Ornithine	128	C1–C5	0%	0%	3%	8%	86%	3%				
Phenylalanine	178	C1–C9	0%	0%	0%	0%	0%	2%	3%	12%	79%	4%
Proline	128	C1–C5	1%	0%	2%	5%	89%	3%				
Pyroglutamate	84	C1–C4	0%	0%	2%	6%	92%					
Pyruvate												
Serine	100	C1–C3	2%	5%	88%	5%						
Succinate	115	C1–C4	2%	1%	2%	24%	72%					
Threonine	115	C1–C4	2%	7%	89%	2%	0%					
Tryptophan	130	C1–C9	5%	0%	6%	0%	4%	1%	0%	7%	13%	65%
Tyrosine	236	C1–C9	5%	1%	0%	0%	1%	0%	1%	3%	9%	80%
Valine	130	C1–C5	3%	0%	2%	4%	87%	2%				
*α*-Ketoglutarate	115	C1–C4	1%	0%	0%	6%	93%					
*β*-alanine	88	C1–C3	6%	89%	3%	2%						
			M	M+1	M+2∼13	M+14	M+15	M+16	M+17	M+18		
Oleate	296	C	6%	1%	0%	1%	3%	12%	14%	62%		

Ion cluster is a group of ions which belong together, all peaks have the same molecular composition but with different isotopes of the carbon atoms. Carbon fragment indicates the number of carbon atoms in a given ion cluster. M is the main molecular ion peak of a metabolite. M+1 is 1 m z^−1^ higher than the M.

## Discussion

We have detected a global upregulation of central carbon metabolism when *C. albicans* hyphal formation was suppressed by high concentrations of the quorum sensing molecule, phenylethyl alcohol. This result is in accordance with our previous two metabolomic studies of *C. albicans* morphogenesis, in which we also observed a global upregulation of central carbon metabolic pathways when hyphal formation was suppressed by farnesol, or a general metabolic downregulation when hyphal growth was promoted by various growth media [14], [15]. The latter observation was further validated by quantifying intracellular ATP levels, which confirmed a lower ATP concentration when hyphal growth was promoted by various growth media (14).

**Figure 7:**
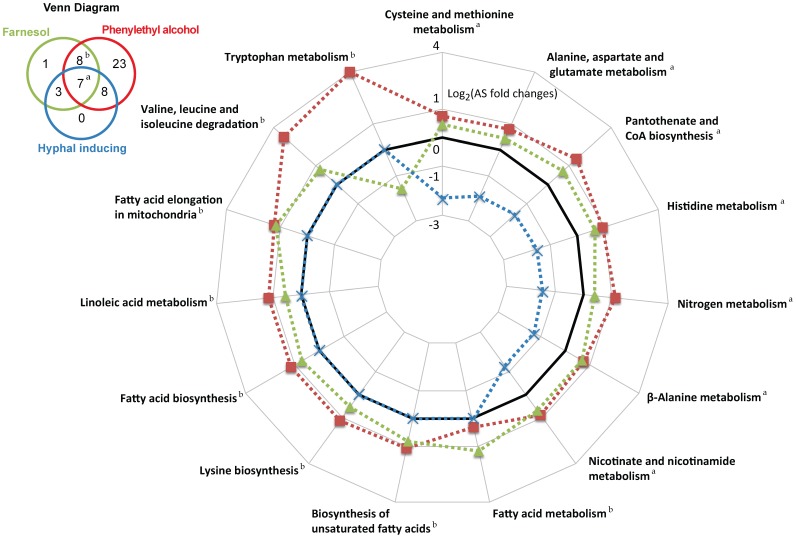
The up- and down-regulation of *C. albicans* metabolic pathways when hyphal growth was suppressed by farnesol, or phenylethyl alcohol, or induced by various growth conditions. Metabolic pathways responding to phenylethyl alcohol (red) are derived from Fig. 5. Metabolic pathways responding to farnesol (green) and hyphae-inducing conditions (blue) are derived from previous studies [14], [15]. A small Venn diagram is displayed to illustrate the unique and common metabolic pathways affected by culturing the cells in the presence of farnesol, phenylethyl alcohol or under various hyphae-inducing conditions. ^a^ indicates the metabolic pathways that respond in common to all three conditions. ^b^ indicates the metabolic pathways that differ from control cells in response to both farnesol and phenylethyl alcohol. The metabolic activities relative to their corresponding controls (black line set to 0) have been plotted using a log_2_ scale. A positive value indicates that a metabolic pathway was up-regulated in comparison to the control. A negative value means the metabolic pathway activity was down-regulated when compared to the control. Only the metabolic pathways with statistically significant (p-value<0.05) changes in activity are shown.

In an attempt to understand the downstream molecular mechanism of *C. albicans* morphogenesis, we compared the metabolic profiles of *C. albicans* cells when growing in the presence of phenylethyl alcohol with the profiles obtained in our previous studies: exposure to farnesol and hyphae-inducing conditions [14], [15]. This permitted us to short-list 7 metabolic pathways that appear to be altered in all three studies (Figure 7). These metabolic pathways encompassed alanine, asparate and glutamate metabolism; *β*-alanine metabolism; cysteine and methionine metabolism; histidine metabolism; nitrogen metabolism; nicotinate and nicotinamide metabolism; and pantothenate and CoA biosynthesis. Therefore, we believe that these seven metabolic pathways are likely to be closely associated with the morphogenetic process of *C. albicans*.

Furthermore, QSMs such as phenylethyl alcohol and farnesol at relatively high concentrations (1–15 mM) seem to influence the lipid metabolism of *C. albicans* independently from morphological changes (Figure 7). We have detected five metabolic pathways related to lipid metabolism that appeared to be significantly upregulated only in the presence of phenylethyl alcohol and farnesol [15]. Phenylethyl alcohol at concentrations between 60 and 140 mM is known to exhibit antimicrobial effects against bacteria (e.g. *Escherichia coli*, *Staphylococcus aureus*, and *Enterococcus faecium*) [16], [17] and fungi (e.g. *C. albicans* itself, *Saccharomyces cerevisiae, Kluyveromyces marxianus,* and *Candida dubliniensis*) [18], [19]. One of its proposed antimicrobial mechanisms is alterations in membrane functions such as permeabilisation of the cell envelope and leakage of potassium ions [16], [17]. Therefore, the upregulation of fatty acid metabolism in response to the presence of phenylethyl alcohol may support our previous hypothesis [15] that *C. albicans* changes its membrane composition in order to reduce the antimicrobial effects of QSMs at high concentrations. In addition, *C. albicans* is likely to benefit from secreting these toxic QSMs during inter-species competition.

**Figure 8:**
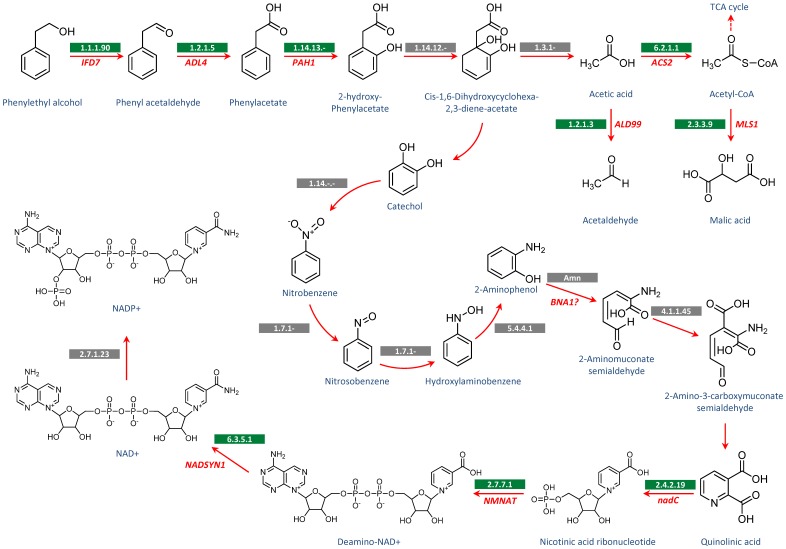
The proposed catabolism of phenylethyl alcohol in *C. albicans*. The pathways illustrate how phenylethyl alcohol could be metabolized in *C. albicans* based on isotope-labelling results. The *C. albicans* genes annotated as encoding particular enzymatic activities are in red text and their corresponding enzyme codes are in green boxes. The genes and enzymes are as follows: *IFD7* (benzyl alcohol dehydrogenase), *ADL4* (aldehyde dehydrogenase), *PAH1* (oxireductase acting on the CH-OH group of donors and incorporation of one atom of oxygen), *ACS2* (acetyl-CoA synthetase), *ALD99* (aldehyde dehydrogenase), *MLS1* (malate synthase), *BNA1* (3-hydroxyanthranilic acid dioxygenase), *nadC* (nicotinate-nucleotide pyrophosphorylase), *NMNAT* (nicotinamide mononucleotide adenylyltransferase), *NADSYN1* (NAD synthetase 1). Enzymes not yet described in *C. albicans* are in grey boxes. The putative enzymes are as follow: 1.12.12- (oxireductase acting on the CH-OH group of donors and incorporation of two atoms of oxygen), 1.3.1- (oxireductase acting on the CH-CH group of donors), 1.14.-.- (oxireductase), 1.7.1.- (nitrobenzene nitroreductase), 5.4.4.1 (hydroxylaminobenzene mutase), Amn (2-aminophenol-1,6-dioxygenase), 4.1.1.45 (aminocarboxymuconate-semialdehyde decaborxylase), 2.7.1.23 (NAD+ kinase).

On the other hand, phenylethyl alcohol appears not only to act as a signalling molecule inducing gene expressions as reported before [6], but it is also taken up from the extracellular medium and metabolised intracellularly as demonstrated by our results. When we provided labelled cells with unlabelled phenylethyl alcohol we observed that almost all amino acids belonging to the histidine, serine, pyruvate, and glutamate families, as well as lactate and glyoxylate, incorporated at least one carbon from phenylethyl alcohol (Figure 6 and Table 3). However, it was a surprise to find the majority of unlabelled-carbon atoms ending up in NAD^+^/NADH and NADP^+^/NADPH molecules. Therefore, we hypothesized that once taken up by *C. albicans* cells, high concentrations of phenylethyl alcohol induce its oxidation back to its known precursor, phenyl acetaldehyde, which is catalysed by a benzyl alcohol dehydrogenase [20] (Figure 8). Although this enzyme has not been described in *C. albicans* yet, we have found a putative benzyl alcohol dehydrogenase gene (*IFD7*) based on orthologous gene identification from the *C. albicans* genome sequence (*C. albicans* SC5314, orf19.629). Phenylacetaldehyde could then be converted into phenylacetate and subsequently into 2-hydroxy-phenylacetate [21] (Figure 8). Moreover, we speculate that 2-hydroxy-phenylacetate could be broken down into catechol and acetic acid via a cis-1,6-dihydroxycyclohexa-2,4-diene-1-acetate intermediate (Figure 8). Similar reactions have been described in bacteria, and are catalysed by oxireductases acting on CH-OH groups with incorporation of one or two atoms of oxygen [21], [22]. Aerobically, acetate would be converted into acetyl-CoA via acetyladenilate, feeding into the TCA cycle and lipid biosynthesis or converted into malate. However, acetate could also be reduced to acetaldehyde via *C. albicans’* aldehyde dehydrogenase (encoded by *ALD99*). Although we believe the reduction of acetate into acetaldehyde would be less favoured under aerobic conditions, it may explain how some threonine molecules were found to be labelled with two ^12^C-atoms, because this amino acid can be synthesised from glycine and acetaldehyde via threonine aldolase (*GLY12*) in *C. albicans*. But, we cannot explain why we did not detect much isotope-labelling in the TCA cycle intermediates considering that some amino acids from the glutamate family were found to contain ^12^C-carbons. We can only speculate that the high carbon flux throughout the central carbon metabolism pathways must have diluted the ^12^C-isotopes to concentrations below the detection limits of our GC-MS method, whilst some amino acids would have a lower turnover inside the cell allowing the detection of their ^12^C-atoms.

Furthermore, we speculate that the benzene ring from phenylethyl alcohol could be incorporated into nucleotide molecules through its metabolic conversion into the pyridine ring of NADH and subsequently NADH is phosphorylated to form NADPH (Figure 8). Based on the current understanding of NADH biosynthesis, there are three possible metabolic routes by which a pyridine ring of NADH could be potentially synthesized: i) via *de novo* synthesis from aspartate, but aspartate was not found to be labelled to any great extent; ii) via salvage pathways by recycling of compounds containing nicotinamide, but phenylethyl alcohol does not contain nicotinamide; or iii) via *de novo* synthesis from tryptophan catabolism. There is no evidence that a benzene ring from phenylethyl alcohol could be directly converted into pyridine through biochemical reactions. One possibility would be for *C. albicans* to convert catechol into 2-aminophenol. Some bacteria have been shown to be able to do this through bezene nitroreductase reactions [23] (Figure 8). *C. albicans* has a dioxygenase that is capable of opening the benzene ring from 3-hydroxyanthranilic acid, a similar molecule to 2-aminophenol, through the incorporation of two atoms of dioxygen and spontaneously rearranging the molecule into a pyridine structure (Figure 8). For example, in the *C. albicans* tryptophan catabolism pathway, 3-hydroxyanthranilate dioxygenase (*BNA1*) catalyses the cleavage of the benzene ring from 3-hydroxyanthranilate into 2-amino-3-carboxymuconate semialdehyde [24], [25]. This unstable compound spontaneously cyclises to form a pyridine structure and becomes quinolinate, an intermediate involved in the *de novo* biosynthesis of NADH from tryptophan. Therefore we speculate that the *C. albicans* 3-hydroxyanthranilate dioxygenase could potentially accept 2-aminophenol as substrate converting it into 2-aminomuconate semialdehyde, which could be converted into 2-amino-3-carboxymuconate semialdehyde via the aminocarboxymuconate-semialdehyde decarboxylase reverse reaction (Figure 8). Aminocarboxymuconate-semialdehyde decarboxylase (EC. 4.1.1.45) has been described in different mammals and bacteria, and plays an important role in tryptophan catabolism [http://www.brenda-enzymes.info/php/result_flat.php4?ecno=4.1.1.45]. The enzyme regulates NADH biosynthesis from amino acids, directly affecting quinolinate and picolinate formation [26]. This enzyme has very low Km values (0.001 to 0.09), which suggest that its reaction should be reversible [http://www.brenda-enzymes.info/php/result_flat.php4?ecno=4.1.1.45]. However, we would expect the cells to have some pyridine ring unlabelled due to *de novo* synthesis, but they did not. Thus, the fully labelled pyridines of cofactors remain puzzling and require further investigations.

An alternative hypothesis regarding the isotope labelling results is that *C. albicans* assimilated ^12^C-carbons by fixing exogenous CO_2_. *S. cerevisae* has been shown experimentally to fix CO_2_ into phosphoenolpyruvate by phosphoenolpyruvate carboxykinase (Pck1p) forming oxaloacetate, an intermediate of the TCA cycle [27], [28]. *C. albicans* often grows within the host in an environment where the CO_2_ level (5%) is more than 150-fold greater than in the atmosphere (0.033%) [29]. The ability to utilise additional carbon from CO_2_ for biosynthetic purposes during morphogenesis, seems to be a good growth and invasion strategy, considering that an atmosphere containing 5 to 15% of CO_2_ also stimulates germ tube formation in *C. albicans* [30], [31]. However, there is no direct evidence that *C. albicans* is capable of fixing CO_2_ for cellular growth, even though *C. albicans* does have a putative phosphoenolpyruvate carboxykinase gene (*PCK1*) based on orthologous gene identification from its genome sequence. Nevertheless, this could explain why a large proportion of lactate molecules were labelled with one ^12^C carbon.

By combining the fact that phenylethyl alcohol could be potentially catabolised mainly into the pyridine structure of NADH/NADPH molecules with the pathways we have previously short-listed as potentially related to the morphogenetic process, we have identified two candidate primary metabolic pathways - nitrogen metabolism and nicotinate/nicotinamide metabolism - which could play a central role in *C. albicans* morphogenesis. These two pathways not only significantly change their activity during various hyphal-inducing conditions as indicated by our previous metabolomics studies, but they are also responsible for the biosynthesis and replenishment of NADH and NADPH molecules in the cell. Nitrogen metabolism plays a role in maintaining the redox balance between the reduced and oxidized states of NADH and NADPH. Nicotinate and nicotinamide metabolism produces NAD^+^ via reutilizing compounds containing nicotinamide, and by *de novo* synthesis of NAD^+^ from tryptophan or aspartate. Subsequently, NAD^+^ can be phosphorylated into NADP^+^ by NAD^+^ kinase [32]. It is important to note that NADH and NADPH have a significant influence on central carbon metabolism. NADH not only acts as an electron carrier mediating energy metabolism, it is also involved in other cellular process such as post-translational modifications that affect the activity of metabolic enzymes. NADPH is a potent reducing agent that drives biochemical reactions toward anabolic metabolism including biosynthesis of lipids, nucleic acids, and amino acids. Therefore, nitrogen metabolism and nicotinate/nicotinamide metabolism have great potential to be the main link between signalling pathways and downstream primary metabolism during *C. albicans* morphogenesis.

### Conclusions

This is the first investigation of the metabolic response of *C. albicans* to phenylethyl alcohol. We observed a global upregulation of central carbon metabolism when filamentous growth was suppressed by phenylethyl alcohol, and we demonstrated that this QSM is not only secreted by *C. albicans* cells, but it is also actively taken up and catabolised intracellularly. We found strong evidence that phenylethyl alcohol is catabolised mainly into the pyridine ring of NADH and NADPH molecules which could affect the concentration of these molecules inside the cells as well as their redox state; explaining its marked effect on the central carbon metabolism of *C. albicans* and consequent inhibition of hyphal formation. NADH and NADPH are important electron carriers responsible for the redox balance of the cell. The disruption of the cell’s redox balance would have major effects on primary metabolism. Therefore, our studies suggest that nitrogen metabolism and nicotinate/nicotinamide metabolism play an important role in the morphogenetic process of *C. albicans,* and further investigations are required to better understand how these pathways are connected to the upstream signalling pathways of morphogenetic regulation in *C. albicans*.
