# Correction: Metabolic Response of *Candida albicans* to Phenylethyl Alcohol under Hyphae-Inducing Conditions

**DOI:** 10.1371/annotation/d395f7a5-ac0a-41e2-abd8-fa947717f6a3

**Published:** 2013-08-22

**Authors:** Ting-Li Han, Sergey Tumanov, Richard D. Cannon, Silas G. Villas-Boas

The legends of Figures 1 and 2 were swapped. The correct version of those figure legends are available below:

**Figure 1. Growth curves of C. albicans cells grown in the presence (red) or absence (blue) of phenylethyl alcohol (15 mM).**

**Figure 2. The morphology of C. albicans cells incubated in the presence of different concentrations of phenylethyl alcohol in minimal mineral medium (MM) at 37°C for 12 h.**

The Images were taken by Nomarksi contrast microscopy with 800× magnification. 

